# 

**Published:** 1891-03-21

**Authors:** 


					WIN BURNER u ^ ISINGLASS
FOR INFANTS 1 n 4 and invalids.
WITH EXTRA NURSING SUPPLEMENT.
Saturday,
March 21st, 1891.
[One Penny.

				

